# ICF rehabilitation set responsiveness after total knee arthroplasty: a retrospective study

**DOI:** 10.3389/fresc.2026.1784781

**Published:** 2026-03-10

**Authors:** Dong Wang, Long Xu, Xin Wang, Yujiao He, Jinlong Cui, Shuhua Zhou, Yin Tong, Feng Gao, Huijun Du, Wei Fan

**Affiliations:** 1 Department of Rehabilitation Medicine, Xiangya Boai Rehabilitation Hospital, Changsha, Hunan, China; 2 Taihe Hospital, Hubei University of Medicine, Shiyan, Hubei, China

**Keywords:** disability and health, international classification of functioning, measurement properties, rehabilitation, responsiveness, total knee arthroplasty

## Abstract

**Introduction:**

The International Classification of Functioning, Disability and Health—Rehabilitation Set (ICF-RS) is a standardized tool for multidimensional rehabilitation assessment, but its responsiveness in post-total knee arthroplasty (TKA) inpatient settings remains underexplored.

**Methods:**

A retrospective single-center cohort study was conducted on 47 patients who underwent primary unilateral TKA and received inpatient rehabilitation in Changsha, China (January 2023–December 2024). ICF-RS scores (30 categories) were assessed at admission and discharge. Responsiveness was evaluated using Wilcoxon signed-rank tests with Benjamini-Hochberg FDR correction. Response counts (improved/stable/worsened) were the primary outcome presentation.

**Results:**

Of 30 ICF-RS categories, 27 showed 100% data completeness; 3 social/sexual items had substantial missingness. Following rehabilitation, 23 categories demonstrated statistically significant improvement after FDR correction. Consistent directional improvement was observed in mobility (d450 Walking: 24/47 improved), self-care (d510–d540), and pain (b280: 26/47 improved) domains. The total score decreased from 47.51 ± 13.59 to 35.81 ± 11.38 (Cohen's d = 1.51). Notably, b710 (joint mobility) showed no responsiveness (0/47 improved, 1/47 worsened), despite expected clinical range-of-motion gains.

**Discussion:**

ICF-RS demonstrates responsiveness to post-TKA inpatient rehabilitation for most functional domains. The lack of change in b710 highlights a limitation of generic assessment tools for capturing localized physical impairments in single-joint conditions, suggesting the need for complementary condition-specific measures. The study context—where ICF-RS scores influence reimbursement—underscores the importance of assessment integrity considerations in function-based payment implementations.

## 1 Introduction

Total knee arthroplasty (TKA) is a primary intervention for end-stage knee osteoarthritis, with surgical volumes rising rapidly in China and globally (1, 2). While surgical techniques have advanced, postoperative functional recovery remains the ultimate indicator of success (3–6). Optimizing assessment tools to capture this recovery is crucial, particularly for aligning rehabilitation with value-based care models (7). However, traditional outcomes often focus on isolated physical parameters (8), lacking a unified framework to capture the multidimensional recovery of patients and to relate these gains to healthcare resource consumption.

The International Classification of Functioning, Disability and Health—Rehabilitation Set (ICF-RS) offers a standardized language to bridge this gap (9, 10). While the ICF-RS has shown promise in diverse populations (11–13), its application in TKA rehabilitation—a short-term, single-joint, and highly standardized process—remains underexplored. Specifically, the responsiveness of generic ICF-RS categories to the rapid, localized functional changes typical of post-TKA recovery, and its integration into payment reforms (e.g., the “Changsha Model”), lacks pilot data.

Furthermore, the integration of functional assessment into health policy adds a new dimension to this inquiry. In Changsha, China, a pilot value-based payment reform has integrated ICF-RS scores into the medical insurance reimbursement system (14, 15). This “natural policy context” provides a unique opportunity to describe the economic profile of rehabilitation under a function-driven payment model.

This pilot retrospective study aimed to address these descriptive gaps. Specifically, we aim to (1): describe the feasibility of ICF-RS in post-TKA inpatients and their baseline impairment profiles (2); assess the internal responsiveness (statistical sensitivity to change) of ICF-RS categories during inpatient rehabilitation; and (3) provide a descriptive cost profile and calculate the “cost per ICF-RS point improvement” as a preliminary efficiency metric. This study does not seek to evaluate the causal effectiveness of the payment reform but rather to provide empirical baseline parameters for future large-scale health economic studies.

## 2 Materials and methods

### 2.1 Study design and setting

This was a retrospective cohort study conducted at Xiangya Boai Rehabilitation Hospital, a tertiary rehabilitation center in Changsha, Hunan Province, China. The reporting follows the STROBE guidelines for observational studies. The hospital serves as a designated institution under Changsha's ICF-RS-based value-based payment system. The study was approved by the hospital's Ethics Committee (Approval No. 2023-15), and the requirement for informed consent was waived due to the retrospective nature of the study.

### 2.2 Participants

Patients were included if they (1): underwent primary unilateral total knee arthroplasty (2); were admitted for first-time post-TKA inpatient rehabilitation (3); were medically stable; (4) were covered by Changsha's medical insurance; and (5) had complete ICF-RS assessments. Patients were excluded if they had bilateral TKA, revision surgery, severe complications, or incomplete data.

From January 2023 to December 2024, all consecutive patients meeting criteria were analyzed (*n* = 47). Demographic and clinical characteristics are presented in Table 1.

**Table 1:** Demographic and clinical characteristics of patients (*n* = 47).

Characteristic	Value
Age, years, median (IQR)	68.00 (61.00, 75.00)
Sex, *n* (%)	
Male	8 (17.0)
Female	39 (83.0)
BMI, kg/m^2^, median (IQR)	25.12 (22.89, 27.34)
Surgery-rehabilitation interval, days, median (IQR)	8.00 (3.00, 29.00)
Length of hospital stay, days, median (IQR)	21.00 (10.00, 27.00)

IQR, interquartile range (25th–75th percentile); BMI, body mass index. Surgery-rehabilitation interval, days from primary TKA surgery to admission to the rehabilitation ward.

### 2.3 Rehabilitation intervention

All patients received a standardized, multidisciplinary rehabilitation protocol based on institutional clinical pathways for post-TKA rehabilitation. The protocol was delivered by a team including physiatrists, physical therapists, occupational therapists, traditional Chinese medicine practitioners, and rehabilitation nurses. Interventions were provided daily or as specified throughout hospitalization.

Physical therapy focused on restoring knee range of motion, muscle strength, and functional mobility through active-assisted and active range of motion exercises, progressive resistance training, and neuromuscular re-education. Functional training included progressive weight-bearing gait training with assistive devices, stair climbing, and transfer training. Physical modalities such as transcutaneous electrical nerve stimulation and therapeutic ultrasound were used for pain and edema management. Treatment frequency was 60 min per day, 6 days per week.

Occupational therapy focused on activities of daily living training, including dressing, bathing, toileting, and household activities. Adaptive techniques and equipment were introduced as needed, and home safety assessment and modification recommendations were provided before discharge.

Traditional Chinese medicine interventions included acupuncture (16) targeting local and distal points to promote circulation and reduce pain (30 min per session, 5 sessions per week), moxibustion for warming meridians and strengthening constitution, and individualized herbal decoctions based on syndrome differentiation. Rehabilitation nursing included patient education on exercise techniques, pain management, fall prevention, and self-care strategies, with continuous monitoring of pain levels and surgical site status.

Detailed treatment protocols are provided in the Supplementary Materials.

### 2.4 Outcome measures

The primary outcome was functional status assessed using the International Classification of Functioning, Disability and Health—Rehabilitation Set (ICF-RS), Chinese version. ICF-RS is a standardized assessment tool comprising 30 categories across three domains: body functions (9 categories), activities (14 categories), and participation (7 categories) (Supplementary Table S1). ICF-RS assessments were conducted at two time points: within 24 hours of admission to the rehabilitation ward (T0) and on the day before or the day of discharge (T1).

All assessments were performed by certified rehabilitation assessors (*n* = 2) from the hospital's Rehabilitation Assessment Department, who were independent from the treating clinical team and blinded to the study objectives. Assessors completed standardized training (40 h) conducted jointly by the Changsha Medical Insurance Bureau and Hunan Rehabilitation Medicine Association (17). All assessors passed a qualification examination demonstrating inter-rater reliability (intraclass correlation coefficient >0.85). Each ICF-RS category is rated on a 5-point scale: 0 = no impairment, 1 = mild impairment (5%–24% limitation), 2 = moderate impairment (25%–49% limitation), 3 = severe impairment (50%–95% limitation), and 4 = complete impairment (96%–100% limitation). Scores of 8 (not applicable) or 9 (not specified) were recorded when the category could not be assessed and were excluded from statistical analysis.

The secondary outcome was hospitalization costs, extracted from the hospital information system at discharge and categorized according to the Chinese national health expenditure classification (18): rehabilitation treatment fees, bed fees, medication fees, examination fees, consultation fees, laboratory fees, nursing fees, material fees, and other fees. To describe efficiency, the “cost per ICF-RS point improvement” was calculated as total cost divided by the change in ICF-RS total score. This metric is a descriptive parameter for the value-based model context and is not a formal cost-effectiveness ratio.

### 2.5 Data collection procedures

Demographic and clinical data (age, sex, body mass index, surgical date, admission and discharge dates) were extracted from electronic medical records by trained research staff using a standardized data collection form. ICF-RS assessment data were retrieved from the Rehabilitation Assessment Department's secure database. Cost data were obtained from the hospital's financial information system. All data were de-identified before analysis, and data accuracy was verified through double data entry and cross-validation between different data sources.

### 2.6 Statistical analysis

All statistical analyses were performed using SPSS version 25.0 (IBM Corp., Armonk, NY, USA), with two-sided *P* < 0.05 considered statistically significant. Continuous variables were tested for normality using the Shapiro–Wilk test. Normally distributed continuous variables are reported as mean ± standard deviation; non-normally distributed continuous variables as median (interquartile range). Categorical variables are reported as frequency (percentage).

For the analysis of functional disability profiles, baseline ICF-RS scores were summarized descriptively, with each category classified as “no or mild impairment” (scores 0–1) or “significant impairment” (scores 2–4). Feasibility was operationalized as data completeness (percentage of valid 0–4 scores) and floor/ceiling effects, defined following ICF conventions (3) where ceiling effect = ≥ 15% of patients scoring 0 (best function) and floor effect = ≥ 15% scoring 4 (worst function).

For the analysis of responsiveness, given that the total ICF-RS score is derived from summing ordinal qualifiers, we performed a sensitivity analysis using the Wilcoxon signed-rank test alongside the paired *t*-test to verify robustness of findings. Admission vs. discharge scores were compared using Wilcoxon signed-rank tests for individual categories (non-normally distributed) and paired t-tests for total score (normally distributed). Effect sizes were calculated as matched-pairs rank-biserial correlation (r_rb) = (R + −R−)/(R + + R−) for non-parametric tests, and Cohen's d for parametric tests. Effect sizes (absolute values of r_rb: 0.1, 0.3, and 0.5) were interpreted as small, medium, and large effects, respectively. Exact *P*-values were used for samples with N_eff ≤25; asymptotic approximation for N_eff >25. To control for multiple comparisons across the 30 ICF-RS categories, *P*-values were adjusted using the Benjamini–Hochberg False Discovery Rate (FDR) procedure (*α* = 0.05). Categories with >50% missing data were excluded from FDR adjustment. For responsiveness analysis of individual ICF-RS categories, cases with codes 8 (not applicable) or 9 (not specified) at either admission or discharge were excluded from analysis for that specific category, resulting in reduced effective sample sizes (N_eff) as reported in Table 3. This approach maximized data utilization while ensuring valid paired comparisons. For total score calculation, categories coded as 8 (not applicable) or 9 (not specified) were treated as missing and excluded from the per-patient total; each patient's total score was thus the sum of only those categories with valid (0–4) qualifier scores. The mean number of contributing categories was 28.7 per patient (range: 27–30 out of 30; see Section 3.1 for details). A sensitivity analysis treating missing categories as 0 (i.e., summing all 30 categories) was also performed to verify robustness. Both raw and adjusted *P*-values are reported in Supplementary Table S2.

For individual category analysis, patients were classified as ‘improved’ (score decrease ≥1 point), ‘worsened’ (score increase ≥1 point), or ’stable’ (no score change). Given the discrete ordinal nature of the ICF-RS qualifier scale (0–4, where 0 = no impairment, 1 = mild, 2 = moderate, 3 = severe, 4 = complete impairment), a 1-point change represents the minimal detectable change on this scale and corresponds to a meaningful shift between adjacent severity levels. Patients at ceiling (score 0 at admission) could only worsen or remain stable; patients at floor (score 4 at admission) could only improve or remain stable.

For the analysis of costs, hospitalization costs were summarized by category using median and interquartile range.

### 2.7 Bias risk considerations

This retrospective study was conducted within the context of Changsha's value-based payment pilot (“Changsha Model”), where ICF-RS improvement scores directly influence institutional reimbursement levels. While individual assessors were not informed of specific patient reimbursement tiers, they were aware of the general policy linking functional gains to payment. This creates potential for systematic scoring bias favoring “improvement” documentation. Although inter-rater reliability was certified (ICC > 0.85), this metric measures consistency among raters, not accuracy or freedom from shared directional bias. Additionally, the absence of a non-intervention control group precludes separation of rehabilitation effects from natural post-surgical recovery. These methodological limitations are addressed further in the Discussion.

## 3 Results

A total of 47 patients who underwent primary unilateral total knee arthroplasty were included in this retrospective cohort study. Their demographic and clinical characteristics, including median age of 68.00 years (IQR 61.00–75.00), predominantly female sex (83.0%), and median length of hospital stay of 21.00 days (IQR 10.00–27.00), are summarized in Table 1.

### 3.1 Feasibility and functional disability profile

Feasibility was operationalized as data completeness and score distributions. For the 30-item ICF-RS, 27 of the 30 categories showed 100% valid data (0–4 scores). Significant missing data (scored as 8 or 9) were observed only in three domains: b640 Sexual functions (*n* = 41, 87.2% missing—patients declined to answer or assessors marked as not applicable due to cultural sensitivity in this acute orthopedic setting), d770 Intimate relationships (*n* = 9, 19.1%), and d850 Remunerative employment (*n* = 8, 17.0%—reflecting the retired status of most patients). These findings suggest high feasibility for core physical and ADL-related rehabilitation metrics but limited applicability of social/sexual items in this older adult cohort (median age 68).

Analysis of score distributions at baseline revealed distinct floor and ceiling effects (Table 2). “Ceiling effects” [≥15% of patients scoring 0 (no impairment); threshold per McHorney & Tarlov, 1995] were observed in 15 categories, most notably in d770 Intimate relationships (80.9%), d550 Eating (74.5%), and b152 Emotional functions (51.1%), indicating that these functions were largely intact at admission. Conversely, “Floor effects” [≥15% scoring 4 (complete impairment)] were prominent in d455 Moving around (89.4%), d640 Doing Housework (48.9%), and d470 Using transportation (36.2%), reflecting the severe mobility restrictions inherent to the acute post-operative phase.

**Table 2:** Baseline ICF-RS score distributions showing floor and ceiling effects (*n* = 47).

Domain	Category	Score 0 (Ceiling), *n* (%)	Score 4 (Floor), *n* (%)
Body functions	b130 Energy/drive	5 (10.6)	0 (0.0)
	b134 Sleep	1 (2.1)	3 (6.4)
	b152 Emotional	24 (51.1)	0 (0.0)
	b280 Pain	0 (0.0)	3 (6.4)
	b455 Exercise tolerance	0 (0.0)	4 (8.5)
	b620 Urination	22 (46.8)	0 (0.0)
	b710 Joint mobility	3 (6.4)	0 (0.0)
	b730 Muscle power	14 (29.8)	0 (0.0)
Activities	d410 Body position change	0 (0.0)	8 (17.0)
	d415 Body position maintain	0 (0.0)	0 (0.0)
	d420 Transferring	23 (48.9)	0 (0.0)
	d450 Walking	20 (42.6)	0 (0.0)
	d455 Moving around	0 (0.0)	42 (89.4)
	d465 Moving w/ equipment	19 (40.4)	0 (0.0)
	d470 Using transportation	0 (0.0)	17 (36.2)
	d510 Washing	12 (25.5)	5 (10.6)
	d520 Body care	15 (31.9)	2 (4.3)
	d530 Toileting	16 (34.0)	0 (0.0)
	d540 Dressing	14 (29.8)	1 (2.1)
	d550 Eating	35 (74.5)	0 (0.0)
	d570 Health maintenance	8 (17.0)	0 (0.0)
	d640 Housework	3 (6.4)	23 (48.9)
Participation	d230 Daily routine	5 (10.6)	6 (12.8)
	d240 Handling stress	5 (10.6)	1 (2.1)
	d660 Assisting others	0 (0.0)	13 (27.7)
	d710 Interpersonal	19 (40.4)	0 (0.0)
	d770 Intimate relationships	38 (80.9)	0 (0.0)
	d850 Employment	24 (51.1)	3 (6.4)
	d920 Recreation	2 (4.3)	7 (14.9)

Ceiling effect: ≥15% scoring 0; Floor effect: ≥15% scoring 4 (bold). b640 (Sexual functions) excluded due to >50% missing data.

Baseline ICF-RS assessments identified significant functional impairments across all three domains. In the body functions domain, universal impairment was observed in b455 exercise tolerance functions (100.00%) and near-universal in b280 sensation of pain (95.74%).

In the activities domain, three categories showed universal significant impairment (100.00%): d410 changing basic body position, d415 maintaining a body position, and d455 moving around. High prevalence was also observed in d640 doing housework (82.61%), d510 washing oneself (61.70%), d520 caring for body parts (61.70%), d240 handling stress and other psychological demands (57.45%), d570 looking after one's health (53.19%), and d540 dressing (53.19%). Lower prevalence was found in d530 toileting (31.91%), d465 moving around using equipment (27.66%), d450 walking (25.53%), and d420 transferring oneself (12.77%). Eating (d550) showed no significant impairment.

In the participation domain, the highest prevalence of significant impairment was found in d660 assisting others (91.49%), d470 using transportation (87.23%), and d920 recreation and leisure (82.98%), followed by d230 carrying out daily routine (65.96%). Lower prevalence was observed in d710 basic interpersonal interactions (25.53%) and d850 remunerative employment (23.08%). Intimate relationships (d770) showed no significant impairment.

### 3.2 Changes in ICF-RS scores

The total ICF-RS score decreased significantly from admission to discharge, reducing from 47.51 ± 13.59 to 35.81 ± 11.38 (paired t-test: t = 10.210, *P* < 0.001; Wilcoxon signed-rank test: Z = −5.777, *P* < 0.001), with a large effect size [Cohen's d = 1.51, 95% CI (1.08, 1.93); mean change 11.70 points, 95% CI (9.42, 13.98)]. A sensitivity analysis including missing categories as 0 in the total yielded identical results, confirming that the exclusion of a small number of non-assessed categories did not affect the findings. Of the 30 individual categories assessed, 24 showed statistically significant improvement (unadjusted *P* < 0.05). After FDR correction, 23 categories maintained statistical significance (adjusted *P* < 0.05); Sleep functions (b134) showed borderline improvement (unadjusted *P* = 0.048, adjusted *P* = 0.059). Effect size analysis using rank-biserial correlation revealed consistent directional improvement (r_rb > 0.5) in most responsive categories (Table 3; see Supplementary Table S2 for full details). Notably, r_rb = 1.00 indicates that all patients who changed showed improvement, not that the clinical effect was maximal; this should be interpreted alongside response counts.

**Table 3:** ICF-RS category changes: response counts and statistical significance.

Code	Category	Improved	Stable	Worsened	N_eff	*P*	q_FDR	Sig
b130	Energy/drive	23	22	2	25	<0.001	<0.001	**
b134	Sleep	15	27	5	20	0.048	0.059	ns
b152	Emotional	13	33	1	14	0.008	0.010	*
b280	Pain	26	20	1	27	<0.001	<0.001	**
b455	Exercise tolerance	9	38	0	9	0.003	0.004	*
b620	Urination	5	41	1	6	0.280	0.324	ns
b640	Sexual	0	5	0	0	—	Excl.	—
**b710**	**Joint mobility**	**0**	**46**	**1**	**1**	**0**.**317**	**0**.**354**	**ns**
b730	Muscle power	12	34	1	13	0.002	0.004	*
d230	Daily routine	22	25	0	22	<0.001	<0.001	**
d240	Handling stress	20	26	1	21	<0.001	<0.001	**
d410	Body position change	13	34	0	13	<0.001	0.001	**
d415	Body position maintain	9	38	0	9	0.004	0.005	*
d420	Transferring	19	28	0	19	<0.001	<0.001	**
d450	Walking	24	23	0	24	<0.001	<0.001	**
d455	Moving around	2	44	1	3	1.000	1.000	ns
d465	Moving w/ equipment	17	30	0	17	<0.001	<0.001	**
d470	Using transportation	22	24	1	23	<0.001	<0.001	**
d510	Washing	18	29	0	18	<0.001	<0.001	**
d520	Body care	21	26	0	21	<0.001	<0.001	**
d530	Toileting	18	29	0	18	<0.001	<0.001	**
d540	Dressing	17	29	1	18	<0.001	<0.001	**
d550	Eating	9	38	0	9	0.003	0.004	*
d570	Health maintenance	26	21	0	26	<0.001	<0.001	**
d640	Housework	19	27	0	19	<0.001	<0.001	**
d660	Assisting others	15	31	1	16	0.001	0.001	**
d710	Interpersonal	12	34	1	13	0.003	0.004	*
d770	Intimate relationships	0	37	0	0	—	1.000	—
d850	Employment	2	33	2	4	0.458	0.492	ns
d920	Recreation	18	29	0	18	<0.001	<0.001	**
Total ICF-RS Score	**—**	**—**	**—**	**—**	**47**	**<0**.**001**	**—**	**(t** **=** **10.210; d** **=** **1.51)**

Improved, score decreased (function improved); Worsened, score increased; N_eff, number of non-zero differences (ties excluded); q_FDR, FDR-adjusted *P*-value; Sig, ** (q < 0.01), *(q < 0.05), ns (not significant). b710 highlighted as key finding discussed in text. See Supplementary Table S2 for effect sizes (r_rb) and rank sums (R+/R−).

The bold values in the row 'b710 Joint mobility' highlight a key finding: despite clinical expectation of improvement, this category showed no statistically significant change (*P* = 0.317, ns), with 0 patients improved and 46 stable. The bold values in the row ‘Total ICF-RS Score’ indicate the overall summary statistics (paired *t*-test: t  = 10.210, Cohen's d = 1.51).

It is noteworthy that for category d550 (Eating), while 74.5% of patients showed no impairment at baseline (Ceiling effect), significant improvement was still detected (*r* = 0.44). Analysis of response counts revealed this was driven by 9 patients (19.1%) improving from mild impairment to no impairment, while 38 patients (80.9%) remained stable, accurately reflecting the localized need for assistance in a subset of patients rather than a generalized recovery of eating ability.

### 3.3 Cost structure and cost-efficiency profile

Total hospitalization costs for the 47 patients amounted to ¥561,040.86 (Table 4). Medical insurance pooled payments covered 59.52% (¥333,946.55) of total costs. The largest cost category was rehabilitation fees, comprising 55.59% (¥311,880.50) of total costs, followed by bed fees at 10.85% (¥60,864.00).

**Table 4:** Composition of total hospitalization costs (*n* = 47).

Expense category	Total amount (¥)	Percentage (%)
Rehabilitation fees	311,880.50	55.59
Bed fees	60,864.00	10.85
Medication fees	45,234.12	8.06
Traditional Chinese medicine fees	38,567.80	6.87
Examination fees	32,456.00	5.78
Laboratory fees	28,345.90	5.05
Nursing fees	18,976.54	3.38
Material fees	15,234.00	2.72
Other fees	9,482.00	1.69
**Total costs**	**561,040.86**	**100**.**00**
Medical insurance pooled payment	333,946.55	59.52
Patient out-of-pocket	227,094.31	40.48

Total costs is bolded to indicate the overall total (¥561,040.86, 100.00%), distinguishing it from the individual cost subcategories.

On a per-patient basis, the median total cost was ¥11,534.56 (IQR 6,178.28–16,562.86). The median “cost per ICF-RS point improvement” was ¥1,043.54 (IQR 757.26–1,589.76), with rehabilitation services contributing ¥556.18 (IQR 419.00–739.25) per point (Table 5). This metric serves as a descriptive efficiency indicator for the value-based payment model.

**Table 5:** Per-patient hospitalization costs and cost per ICF-RS point improvement, median (IQR).

Expense category	Median cost per patient (¥)	Cost per point improvement (¥/point)
Total costs	11,534.56 (6,178.28, 16,562.86)	1,043.54 (757.26, 1,589.76)
Medical insurance payment	5,888.15 (2,697.44, 11,412.51)	608.49 (378.10, 881.95)
Out-of-pocket expenses	4,274.32 (2,898.06, 5,868.12)	427.09 (197.30, 699.45)
Rehabilitation fees	6,774.00 (3,061.00, 9,393.00)	556.18 (419.00, 739.25)

IQR, interquartile range (25th–75th percentile); Cost per point improvement = total cost/(admission ICF-RS score−discharge ICF-RS score).

## 4 Discussion

### 4.1 Principal findings

This retrospective study demonstrates that the ICF-RS is responsive to functional changes during post-TKA inpatient rehabilitation for most domains. Of the 30 categories assessed, 23 showed statistically significant improvement after FDR correction, with consistent directional improvement in mobility-related (d450 Walking: 24/47 improved), self-care (d510-d540: 17–21/47 improved), and pain domains (b280: 26/47 improved) (19). The total ICF-RS score decreased from 47.51 ± 13.59 to 35.81 ± 11.38 (Cohen's d = 1.51), indicating robust overall responsiveness. Notably, b134 Sleep showed borderline improvement, consistent with previous findings of sleep disturbances after TKA (20). However, in the absence of a control group, these improvements should be interpreted as evidence of responsiveness (statistical sensitivity to change) rather than causal treatment effects.

### 4.2 Generic vs. condition-specific assessment: the b710 divergence

A key finding of this study is the complete lack of responsiveness in b710 (Mobility of joint functions): 0/47 patients showed improvement, 46/47 remained stable, and 1/47 worsened. This is notable because TKA patients typically experience objective improvements in knee range of motion (ROM) during rehabilitation, measurable by goniometry.

This discrepancy highlights a specific property of the ICF-RS coding standards applied in this context. According to the “Assessment Guideline for ICF-RS” (T/CARM 001—2020) used in this study (21), the qualifier for b710 (Mobility of joint functions) is defined by the *number* of affected joints rather than the degree of restriction in a single joint (Score 0: No limitation; Score 1: 1–4 joints limited; Score 2: 5–8 joints limited). Since all participants were unilateral TKA patients without other significant joint impairments, they were classified as having “1 joint limited” (Score 1) at admission. Although their knee range of motion improved substantially during rehabilitation, they remained in the “1–4 joints limited” category at discharge, resulting in a stable score of 1. This “structural stability” makes the generic b710 item effectively unresponsive to improvements in single-joint conditions, essentially functioning as a count of comorbidities rather than a measure of joint function recovery.

This finding has implications for tool selection in TKA rehabilitation research and practice. Huang et al. (2021) developed a TKA-specific ICF Core Set that includes more granular physical impairment categories (22). Our data suggest that when precise measurement of localized physical impairments is required, ICF-RS should be complemented with condition-specific measures (e.g., goniometry, Timed Up and Go test) rather than used as a standalone tool. Similar applications of ICF-RS in other musculoskeletal conditions have reported comparable patterns (23, 24). This trade-off between comprehensiveness (ICF-RS covers 30 domains) and sensitivity (condition-specific tools capture nuanced changes) warrants consideration in future protocol design.

### 4.3 Methodological consideration: assessment integrity in a payment-linked context

A critical methodological consideration is that this study was conducted within Changsha's value-based payment pilot (“Changsha Model”), where ICF-RS functional improvement scores directly influence institutional reimbursement (14, 15). This context creates inherent tension between assessment accuracy and financial incentives.

Several observations from our data warrant discussion. First, regarding directional consistency, in most categories, almost all patients who showed change demonstrated improvement (e.g., d450: 24 improved, 0 worsened). While this pattern is clinically expected during acute post-surgical rehabilitation, it also represents the direction of financial incentive. Second, regarding quality control measures, assessors were certified (ICC > 0.85) and blinded to individual reimbursement tiers. However, ICC measures inter-rater consistency, not accuracy or freedom from shared directional bias. Assessors were aware of the general policy context. Third, regarding floor and ceiling effects, items like d455 (Moving around) showed severe floor effects (89% scored 4 at baseline), limiting measurable improvement. Conversely, items with ceiling effects (e.g., d550 Eating) showed localized improvement driven by small subsets (9/47 patients).

We acknowledge that in retrospective designs without external validation (e.g., blinded independent assessment), we cannot definitively exclude scoring bias. Future prospective studies should consider incorporating blinded assessors and/or objective outcome measures (e.g., accelerometry, gait analysis) to triangulate ICF-RS data.

### 4.4 Descriptive cost profile

In the context of Changsha's value-based payment pilot (25–27), the median total hospitalization cost was ¥11,534.56 (IQR 6,178–16,563), with rehabilitation fees comprising 55.59% of total costs. An exploratory “cost per ICF-RS point improvement” metric was calculated at ¥1,043.54 per point (IQR 757–1,590). These figures provide descriptive baseline parameters for this specific care pathway within the local reimbursement context.

### 4.5 Limitations

First, the single-center design with small sample size (*n* = 47) limits generalizability. Second, the retrospective design with payment-linked assessment creates potential for systematic scoring bias that cannot be fully excluded. Third, without a control group, we cannot separate rehabilitation effects from natural post-surgical recovery. Fourth, item-level analyses (30 comparisons) remain exploratory despite FDR correction, given small sample size and correlated outcomes across ICF-RS domains.

Fifth, regarding the exploratory cost analysis, the “cost per ICF-RS point improvement” metric has significant limitations: (a) there is no control group or historical benchmark for comparison; (b) the cost-per-point value lacks established clinical or economic meaningfulness; (c) the functional improvement denominator may be affected by assessment bias; and (d) cost data spanning 2023–2024 were not adjusted for inflation. These figures should be interpreted as descriptive baseline parameters for the local reimbursement context, not as evidence of cost-effectiveness.

Sixth, this study assessed internal responsiveness (statistical sensitivity to change within individuals over time) only. External responsiveness—the association between ICF-RS score changes and changes in external criterion measures (e.g., patient-reported global improvement, objective functional tests)—was not evaluated. Therefore, while our findings demonstrate that the ICF-RS detects statistically significant changes, conclusions about the clinical meaningfulness of observed improvements cannot be drawn from this study alone.

Seventh, while ICF-RS captures broad functional domains, condition-specific outcomes such as return to athletics (28, 29), kneeling ability (30), psychological adaptation (31, 32), and social participation (33) warrant dedicated assessment in TKA populations to complement generic rehabilitation outcome measures.

Future research should employ prospective designs with blinded assessors, include waiting-list or standard-care control groups, and incorporate objective outcome measures to validate ICF-RS scores in payment-linked contexts. Health economic evaluations should include formal cost-effectiveness analysis with appropriate comparators.

## 5 Conclusion

The ICF-RS demonstrates responsiveness to post-TKA inpatient rehabilitation for most functional domains, supporting its utility as a multidimensional outcome measure. However, the lack of change in b710 (joint mobility) highlights a limitation of generic assessment tools for capturing localized physical impairments in single-joint conditions, suggesting that complementary condition-specific measures may be warranted. The study context—where assessment scores influence reimbursement—underscores the importance of robust quality control mechanisms and prospective validation in future implementations of function-based payment systems.

## Data Availability

The data analyzed in this study is subject to the following licenses/restrictions: the raw data supporting the conclusions of this article will be made available by the authors upon reasonable request, subject to institutional data sharing policies. Requests to access these datasets should be directed to Wei Fan, ot.fanwei@hotmail.com.
