# Supplementary material for: ICF rehabilitation set responsiveness after total knee arthroplasty: a retrospective study
Source: Front Rehabil Sci. 2026 Mar 10;7:1784781. doi: 10.3389/fresc.2026.1784781 (PMC13008864; doi:10.3389/fresc.2026.1784781)
Supplement: Supplementary file 1 [file Table1.docx]

# Supplementary Material

**Manuscript Title**: ICF Rehabilitation Set Responsiveness After Total Knee Arthroplasty: A Retrospective Study

## Table S1. ICF-RS 30 Categories Across Three Dimensions

| Body Functions | Code | Activities | Code | Participation | Code |
| --- | --- | --- | --- | --- | --- |
| Energy and drive functions | b130 | Handling stress | d240 | Carrying out daily routine | d230 |
| Sleep functions | b134 | Changing body position | d410 | Using transportation | d470 |
| Emotional functions | b152 | Maintaining body position | d415 | Assisting others | d660 |
| Sensation of pain | b280 | Transferring oneself | d420 | Interpersonal interactions | d710 |
| Exercise tolerance | b455 | Walking | d450 | Intimate relationships | d770 |
| Urination functions | b620 | Moving around | d455 | Remunerative employment | d850 |
| Sexual functions | b640 | Moving with equipment | d465 | Recreation and leisure | d920 |
| Joint mobility | b710 | Washing oneself | d510 |  |  |
| Muscle power | b730 | Caring for body parts | d520 |  |  |
|  |  | Toileting | d530 |  |  |
|  |  | Dressing | d540 |  |  |
|  |  | Eating | d550 |  |  |
|  |  | Looking after health | d570 |  |  |
|  |  | Doing housework | d640 |  |  |

## Table S2. ICF-RS Item-Level Analysis: Detailed Statistical Results

### Statistical Methodology Notes

- **P-values**: Wilcoxon signed-rank test
- `method='exact'` for N_eff ≤ 25 (recommended for small samples)
- `method='asymptotic'` for N_eff > 25
- **Effect size**: Matched-pairs **rank-biserial correlation (r_rb)**
- Formula: r_rb = (R+ − R−) / (R+ + R−)
- Range: -1 to +1; positive values indicate improvement (score decrease)
- Interpretation: Absolute value of r_rb < 0.3 = small, 0.3–0.5 = medium, > 0.5 = large [ref: Kerby, 2014]
- **Ties handling**: Zero-differences excluded from rank calculation (standard Wilcoxon procedure)
- **Missing data**: Scores of 8 ('not specified') or 9 ('not applicable') excluded from analysis
- **FDR correction**: Benjamini–Hochberg procedure (α = 0.05); items with >50% missingness (b640) excluded

### Full Results Table

| Code | Category | N | Miss% | Impr | Stable | Worse | N_eff | R+ | R− | P | Method | q_FDR | r_rb |
| --- | --- | --- | --- | --- | --- | --- | --- | --- | --- | --- | --- | --- | --- |
| b130 | Energy/drive | 47 | 0.0 | 23 | 22 | 2 | 25 | 302 | 23 | <0.001 | exact | <0.001 | 0.86 |
| b134 | Sleep | 47 | 0.0 | 15 | 27 | 5 | 20 | 156 | 54 | 0.048 | exact | 0.059 | 0.49 |
| b152 | Emotional | 47 | 0.0 | 13 | 33 | 1 | 14 | 94 | 12 | 0.008 | exact | 0.010 | 0.78 |
| b280 | Pain | 47 | 0.0 | 26 | 20 | 1 | 27 | 353 | 25 | <0.001 | approx | <0.001 | 0.87 |
| b455 | Ex. tolerance | 47 | 0.0 | 9 | 38 | 0 | 9 | 45 | 0 | 0.003 | exact | 0.004 | 1.00 |
| b620 | Urination | 47 | 0.0 | 5 | 41 | 1 | 6 | 16 | 6 | 0.280 | exact | 0.324 | 0.48 |
| b640 | Sexual | 5 | 87.2 | 0 | 5 | 0 | 0 | — | — | — | NA | Excl. | — |
| b710 | Joint mobility | 47 | 0.0 | 0 | 46 | 1 | 1 | 0 | 1 | 0.317 | exact | 0.354 | −1.00† |
| b730 | Muscle power | 47 | 0.0 | 12 | 34 | 1 | 13 | 84 | 7 | 0.002 | exact | 0.004 | 0.85 |
| d230 | Daily routine | 47 | 0.0 | 22 | 25 | 0 | 22 | 253 | 0 | <0.001 | exact | <0.001 | 1.00 |
| d240 | Handling stress | 47 | 0.0 | 20 | 26 | 1 | 21 | 220 | 10 | <0.001 | exact | <0.001 | 0.91 |
| d410 | Body pos. change | 47 | 0.0 | 13 | 34 | 0 | 13 | 91 | 0 | <0.001 | exact | 0.001 | 1.00 |
| d415 | Body pos. main. | 47 | 0.0 | 9 | 38 | 0 | 9 | 45 | 0 | 0.004 | exact | 0.005 | 1.00 |
| d420 | Transferring | 47 | 0.0 | 19 | 28 | 0 | 19 | 190 | 0 | <0.001 | exact | <0.001 | 1.00 |
| d450 | Walking | 47 | 0.0 | 24 | 23 | 0 | 24 | 300 | 0 | <0.001 | exact | <0.001 | 1.00 |
| d455 | Moving around | 47 | 0.0 | 2 | 44 | 1 | 3 | 3 | 3 | 1.000 | exact | 1.000 | 0.00† |
| d465 | Moving w/ equip | 47 | 0.0 | 17 | 30 | 0 | 17 | 153 | 0 | <0.001 | exact | <0.001 | 1.00 |
| d470 | Transport | 47 | 0.0 | 22 | 24 | 1 | 23 | 258 | 18 | <0.001 | exact | <0.001 | 0.87 |
| d510 | Washing | 47 | 0.0 | 18 | 29 | 0 | 18 | 171 | 0 | <0.001 | exact | <0.001 | 1.00 |
| d520 | Body care | 47 | 0.0 | 21 | 26 | 0 | 21 | 231 | 0 | <0.001 | exact | <0.001 | 1.00 |
| d530 | Toileting | 47 | 0.0 | 18 | 29 | 0 | 18 | 171 | 0 | <0.001 | exact | <0.001 | 1.00 |
| d540 | Dressing | 47 | 0.0 | 17 | 29 | 1 | 18 | 167 | 4 | <0.001 | exact | <0.001 | 0.95 |
| d550 | Eating | 47 | 0.0 | 9 | 38 | 0 | 9 | 45 | 0 | 0.003 | exact | 0.004 | 1.00 |
| d570 | Health maint. | 47 | 0.0 | 26 | 21 | 0 | 26 | 351 | 0 | <0.001 | approx | <0.001 | 1.00 |
| d640 | Housework | 46 | 2.1 | 19 | 27 | 0 | 19 | 190 | 0 | <0.001 | exact | <0.001 | 1.00 |
| d660 | Assisting others | 47 | 0.0 | 15 | 31 | 1 | 16 | 130 | 6 | 0.001 | exact | 0.001 | 0.90 |
| d710 | Interpersonal | 47 | 0.0 | 12 | 34 | 1 | 13 | 86 | 6 | 0.003 | exact | 0.004 | 0.88 |
| d770 | Intimate relat. | 37 | 19.1 | 0 | 37 | 0 | 0 | — | — | — | NA | 1.000 | — |
| d850 | Employment | 37 | 17.0 | 2 | 33 | 2 | 4 | 7 | 3 | 0.458 | exact | 0.492 | 0.40 |
| d920 | Recreation | 47 | 0.0 | 18 | 29 | 0 | 18 | 171 | 0 | <0.001 | exact | <0.001 | 1.00 |

**Abbreviations**: N = valid cases; Miss% = percentage missing (8/9 scores); Impr = improved; Worse = worsened; N_eff = non-zero differences; R+ = sum of positive ranks; R− = sum of negative ranks; q_FDR = FDR-adjusted P-value; r_rb = rank-biserial correlation.

**Notes**:

- † Items with N_eff ≤ 3: effect size should be interpreted with caution due to extreme sample size; response counts provide more meaningful information.
- r_rb = 1.00 indicates all patients who changed showed improvement (no worsening).
- r_rb = −1.00 (b710) indicates the single patient who changed showed worsening.

### Summary

- **23/30 categories** showed robust significance after FDR correction (q < 0.05)
- **b134 (Sleep)** lost significance after correction (q = 0.059)
- **6 categories** showed no significant change: b620, b640, b710, d455, d770, d850

### Edge Case Explanations

| Code | Explanation |
| --- | --- |
| **d455** | N_eff=3: 2 improved, 1 worsened → R+=3, R−=3 → balanced ranks yield P=1.000 and r_rb=0.00 (no net directional change). Floor effect (89% scored 4 at baseline) limits measurable improvement. |
| **b710** | N_eff=1: Only 1 patient changed (worsened by 1 point) → r_rb=−1.00. This reflects floor effect: most patients already scored 1 ("mild impairment"), leaving no room for improvement in this coarse scale. |
| **b640** | 87% missing: Sexual function was not assessed/applicable for most patients in this acute orthopedic setting. Excluded from FDR correction. |

## Supplementary Methods: Rehabilitation Treatment Protocol

All patients received a standardized, multidisciplinary inpatient rehabilitation protocol following TKA. The protocol was delivered by a team of physiatrists, physical therapists, occupational therapists, traditional Chinese medicine (TCM) practitioners, and rehabilitation nurses.

### Table S3. Summary of Rehabilitation Interventions

| Component | Key Interventions | Frequency |
| --- | --- | --- |
| **Postoperative care** | Multimodal analgesia (acetaminophen, NSAIDs); VTE prophylaxis (enoxaparin + pneumatic compression); cryotherapy and limb elevation | Per protocol |
| **Physical therapy** | ROM exercises, progressive resistance training (quadriceps, hamstrings, hip abductors), gait training with assistive devices, transfer training, stair climbing, stationary cycling | 60 min/day, 6 days/week |
| **Physical agents** | TENS (80–100 Hz, 30 min/session); therapeutic ultrasound (1 MHz, pulsed mode) | As prescribed |
| **Occupational therapy** | ADL training (dressing, bathing, toileting, household tasks); adaptive equipment provision (reacher, sock aid, raised toilet seat); home safety assessment | Daily |
| **Psychological support** | HADS screening at admission; cognitive-behavioral strategies for patients with elevated scores | As indicated |
| **Acupuncture** | Local points (ST34, SP9, SP10, GB34, Ashi) and distal points (ST36); manual stimulation to achieve Deqi; 30 min retention | 1 session/day, 5 days/week |
| **Moxibustion** | Indirect moxibustion at ST36 and SP6; 10–15 min per point | As prescribed |
| **Chinese herbal medicine** | Modified Taohong Siwu Tang; individualized per TCM syndrome differentiation | Twice daily, oral |
| **Rehabilitation nursing** | Pain monitoring (NRS), wound assessment, fall prevention, patient education (exercises, self-care), mobility assistance | Continuous |

### Protocol Adherence

All interventions were documented in the electronic medical record. Treatment attendance and completion rates were monitored by the rehabilitation team. Deviations from the protocol were documented and reviewed in weekly multidisciplinary team meetings.
